# Child psychiatric hospitalizations in the Brazilian public health system: an exploratory study

**DOI:** 10.1590/2237-6089-2019-0064

**Published:** 2020-10-08

**Authors:** Maximiliano Loiola Ponte de Souza, Rossano Cabral Lima

**Affiliations:** 1 Escritório Técnico Fundação Oswaldo Cruz EusébioCE Brazil Grupo Temático de Saúde da Família, Escritório Técnico da Fundação Oswaldo Cruz (FIOCRUZ) no Ceará, Eusébio, CE, Brazil.; 2 Instituto de Medicina Social Universidade do Estado do Rio de Janeiro Rio de JaneiroRJ Brazil Instituto de Medicina Social, Universidade do Estado do Rio de Janeiro (UERJ), Rio de Janeiro, RJ, Brazil.

**Keywords:** Child, hospitalized, psychiatry, mental health, Brazil

## Abstract

**Objective:**

To describe the characteristics and rates of child psychiatric hospitalizations in the Brazilian public health system, as well as their variation according to the country’s macro-regions.

**Methods:**

This was a descriptive study focusing on year 2017, having as main source the Brazilian Ministry of Health’s hospital information system. Child hospitalizations were considered to encompass those of individuals under 13 years of age whose main diagnosis was included in Chapter V of the International Classification of Diseases, 10th edition (ICD-10).

**Results:**

The most prevalent diagnostic group was F10-F19, with 24.1% (21.8-26.3), followed by groups F30-39, F80-F89, F90-F99, F20-F29, with no statistical difference among them. Hospitalizations occurred more frequently in non-psychiatric hospitals, with 93.1% (91.7-94.4); most hospitalizations lasted for up to one week – 75.5% (73.2-77.8). The national hospitalization rate was 4.3 (4.1-4.5)/100,000, showing variations according to macro-regions. The highest rate was found in the South macro-region and the lowest in the Northeast, with values of 10.9 (9.9-12.0)/100,000 and 1.9 (1.6-2.2)/100,000, respectively. Intermediate values were found in the other macro-regions.

**Conclusion:**

Some specificities of the characteristics of hospitalizations for psychiatric reasons in this age group could be attested, as well as important variations in their occurrence among the different macro-regions of the country.

## Introduction

Child mental health is a secondary and late agenda in public health policies in Brazil. Only in the beginning of the last decade did the country begin to contemplate this age group, prioritizing the standardization, implantation and expansion of Child Psychosocial Care Centers (Centro de Atenção Psicossocial Infanto-Juvenil [CAPSi]) – outpatient and community services offered by multi-professional teams to the population living in the assigned coverage area – as the main strategy for expanding mental care among children, especially those with severe and persistent disorders.^1^

Despite the principle of care in Brazilian territory,^1 , 2^ a small group of children with mental problems will require temporary hospitalizations. In Brazil, studies designed to analyze the characteristics of psychiatric hospitalizations either failed to present data by age,^3^ excluded the pediatric population,^4^ or investigated a small number of cases in this age group.^5^ As a result, little is known about child psychiatric hospitalizations in Brazil, their characteristics, dimensions and variations across the national territory – information that is important for the formulation and evaluation of public health policies for this age group. Thus, this article aimed to describe the characteristics and rates of child psychiatric hospitalizations in the Brazilian public health system, as well as their variations according to the country’s macro-regions.

## Method

### Study design and population

This descriptive study focused on year 2017. The population under 13 years old in Brazil, in 2017, estimated based on the official censuses of 2000 and 2010^6^ by means of annual geometric interpolation, was 53,334,085. This population was unequally distributed across the five Brazilian macro-regions: Center-West, 7.5%; North, 11.0%; South, 12.9%; Northeast, 30.7%; Southeast, 37.8%.

### Data source

Data on psychiatric hospitalizations were obtained from the Brazilian Ministry of Health’s hospital information system.^7^ Information on the characteristics of the hospitals in which these hospitalizations occurred were obtained from the National Registry of Health Establishments (Cadastro Nacional de Estabelecimentos de Saúde).^8^

### Inclusion criteria

Psychiatric hospitalizations were considered to encompass all those whose main diagnosis was classified as “mental and behavioral disorders” in Chapter V of ICD-10^9^ and that occurred in Brazil during the study period. Child hospitalizations were those of individuals younger than 13 years.

### Study variables

Psychiatric diagnostic group,^9^ length of hospital stay (0-7 days **,** 8-29 days, 30 days or more), type of hospital (psychiatric or non-psychiatric), and macro-region of residence of the children were the study variables collected.

### Measurements and statistical analyses

Descriptive analysis included the generation of absolute and relative frequencies, expressed as percentages. Hospitalization rates were calculated by the ratio between the number of hospitalizations and the respective population, expressed in hospitalizations/100,000 inhabitants, and standardized by sex and age, using the direct method, having the Brazilian population as reference. For both percentages and rates, 95% confidence intervals (95%CI) were calculated. It was assumed that there would be a statistical difference between the groups when the values did not present intersections considering their confidence intervals.

## Results

There were 1,371 admissions. According to Table 1 , the most prevalent diagnostic group was F10-F19 (mental and behavioral disorders due to the use of psychoactive substance), with 24.1% (21.8-26.3), followed by F30-F39 (mood disorders [affective]), F80-F89 (psychological development disorders), F90-F99 (behavioral disorders and emotional disorders that usually appear during childhood or adolescence), F20-F29 (schizophrenia, schizotypal disorders and delusional disorders); there were no statistical differences among the different diagnoses. Hospitalizations occurred more frequently in non-psychiatric hospitals (93.1% [91.7-94.4]) of the cases, and most of them lasted up to one week (75.5% [73.2-77.8]).

**Table 1:** Child psychiatric hospitalizations* in Brazil in 2017, according to diagnostic group, type of hospital and length of hospital stay (n = 1,371)

	n	%	95%CI
Diagnostic group			
F10-F19	330	24.1	21.8-26.3
F30-F39	185	13.5	11.7-15.3
F80-F89	174	12.7	10.9-14.5
F90-F99	167	12.2	10.4-13.9
F20-F29	138	10.1	8.5-11.7
F70-F79	120	8.8	7.3-10.2
F00-F09	116	8.5	7.0-9.9
F40-F48	98	7.1	5.8-8.5
F60-F69	32	2.3	1.5-3.1
F50-F59	11	0.8	0.3-1.3
Type of hospital			
Psychiatric	95	6.9	5.6-8.3
Non-psychiatric	1,276	93.1	91.7-94.4
Length of hospital stay			
0-7 days	1,035	75.5	73.2-77.8
8-29 days	268	19.5	17.4-21.6
30 days or more	68	5.0	3.8-6.1

95%CI = 95% confidence interval; F00-F09 = organic mental disorders, including symptomatic ones; F10-F19 = mental and behavioral disorders due to the use of psychoactive substances; F20-F29 = schizophrenia, schizotypal disorders and delusional disorders; F30-F39 = mood disorders [affective]; F40-F48 = neurotic disorders, stress-related disorders and somatoform disorders; F50-F59 = behavioral syndromes associated with physiological dysfunctions and physical factors; F60-F69 = personality disorders and adult behavior; F70-F79 = mental retardation; F80-F89 = psychological development disorders; F90-F98 = behavioral disorders and emotional disorders that usually appear in childhood or adolescence.

* Individuals under 13 years of age.

The national hospitalization rate was 4.3 (4.1-4.5)/100,000, with variations according to macro-regions ( Table 2 ). The highest rate was found in the South and the lowest in the Northeast, with values of 10.9 (9.9-12.0)/100,000 and 1.9 (1.6-2.2)/100,000, respectively. Intermediate values were found in the other macro-regions.

**Table 2:** - Child psychiatric hospitalization rates* in Brazil in 2017, according to macro-region (n = 1,371)

	n	Rate^†^	95%CI
Northeast	178	1.9	1.6-2.2
North	114	2,6	1.3-3.7
Center-West	84	3.4	2.8-4.2
Southeast	572	4.9	4.5-5.3
South	423	10.9	9.9-12.0
Brazil	1.371	4.3	4.1-4.5

95%CI = Confidence Interval 95%.

* Individuals under 13 years of age.

^†^ Hospitalizations/100,000 inhabitants.

## Discussion

Considering the currently available literature, this is an unprecedented national epidemiological study on child psychiatric hospitalizations in Brazil. Some specific characteristics of hospitalization in this age group can be evidenced, as well as important disparities in their occurrence among the different regions of the country.

In the absence of a national study on psychiatric hospitalizations in the general population of Brazil, the investigation carried out in the state of Rio Grande do Sul,^3^ in the South macro-region, was used as a comparative proxy. It is noteworthy that Rio Grande do Sul is one of the most prosperous states in the country, with a more structured mental health care network in relation to other Brazilian states.^3 , 4^

Comparing the diagnostic profile of child psychiatric hospitalizations in Brazil with those referring to the general population in the Rio Grande do Sul state,^3^ it was observed that in both scenarios the most prevalent diagnostic group was F10-F19; however, in Rio Grande do Sul, the percentage was higher, namely 33.6%. Our findings show that this diagnostic group is a public health problem already in childhood, and not exclusive to adolescents and adults, demanding specific intervention strategies. In both groups, there was an equal proportion, 8.5%, of cases with the F00-F09 diagnosis, which points to the importance of investigating general medical conditions associated with psychiatric illnesses in all phases of life. Conversely, in the general population of Rio Grande do Sul, hospitalizations for F20-F29 and F30-F39 occurred much more frequently, at 27.6% and 22.0%. These percentages are 2.7- and 1.6-fold higher than the ones recorded for Brazilian children. It is well documented in the literature that, although these diagnostic groups occur in childhood, their prevalence increases with age.^10 , 11^ Finally, the diagnostic groups F80-F89 and F90-F99, which were not mentioned in the hospitalization profile of the general population of Rio Grande do Sul,^3^ accounted for about 25% of all child psychiatric hospitalizations in Brazil, emerging as another specificity of the hospitalization profile of this group.

Most child psychiatric hospitalizations in Brazil (93.1% [91.7-94.4]) were performed in non-psychiatric hospitals. This percentage was 3 times higher than that observed in the general population of Rio Grande do Sul (30.9%), a pioneer state in the implementation of psychiatric hospitalization services in general hospitals.^3^ According to international recommendations, the hospitalization of children, when necessary, should preferably be performed in places other than psychiatric hospitals.^12^

It was also observed that approximately three quarters of the hospitalizations analyzed in the present study lasted for up to one week, with a clear prioritization of brief hospitalizations. International studies involving children and adolescents had already indicated a tendency towards shorter hospitalizations.^13^ In the USA, median length of stay fell from 12.2 days in 1990 to 4.5 days in 2000,^14^ and in Norway, in the age group from 10 to 18 years, the average length of stay was 8.5 days.^15^ For the general population of Rio Grande do Sul, the mean length of psychiatric hospitalization was 16.1 days for hospitalizations due to alcohol and other substances, and 20.0 days for other mental disorders.^4^

Thus, if on the one hand, hospitalization time observed in the Brazilian pediatric population is close to that found in other countries, on the other hand, it is significantly lower than that found for the general national population, indicating the need for studies that may identify the factors associated with this trend. Preliminarily, two possible explanatory paths are raised. First, such a trend could be associated with the higher occurrence of child psychiatric hospitalizations in general or pediatric hospitals: there is abundant evidence showing that hospitalizations in these services tend to be shorter in comparison to those occurring in psychiatric hospitals.^3 , 4^ The second hypothesis would be related to the diagnostic profile of child hospitalizations, characterized by a lower frequency of the F20-F29 and F30-F39 diagnostic groups, which require longer hospitalization time due to, among other factors, the time required for medications to have therapeutic effects.^10 , 11^

The data from this study pointed to higher hospitalization rates in the South macro-region of Brazil, which were, respectively, 2.2, 3.2, 4.2 and 5.7 times higher than those observed in the Southeast, Central-West, North and Northeast macro-regions. Rates were highest in the macro-region with the highest human development index, and lowest in the macro-region with the lowest index.^16^ Statistics on hospital morbidity are strongly influenced by the availability of services.^3 , 4^ Therefore, the differences observed are possibly associated with the availability of a greater range of services in which child psychiatric hospitalizations are carried out in the South macro-region in comparison with the others, especially the Northeast. This hypothesis is reinforced by the fact that most child hospitalizations were performed in non-psychiatric hospitals in the southern states of the country – the South has also been shown to have implemented the greatest expansion of psychiatric hospitalizations in general hospitals in Brazil.^3 , 4^ Further studies are needed to understand the regional variations presented here.

We can conclude that the F10-F19 diagnostic group is already relevant as a cause of hospitalization in children, and that the F80-F89 and F90-F99 diagnostic groups, which are inexpressive in adults, are important in this age group. The vast majority of child psychiatric hospitalizations were performed in non-psychiatric hospitals and lasted less than a week. In addition, there were important variations in child psychiatric hospitalization rates and hospitalizations, possibly expressing regional inequalities in the provision of services.

The data presented here outline a profile, albeit brief, of child psychiatric hospitalizations in Brazil, adding to a scarce national and international literature on the topic and thus opening the way for further research. The advancement of public mental health policies directed to children depends on the availability of epidemiological data from health services in order to qualify crisis intervention, giving priority to care in territorial devices and beds in pediatric hospitals.
